# C3-glomerulonephritis in New Zealand – a case series

**DOI:** 10.1186/s12882-020-02056-5

**Published:** 2020-09-17

**Authors:** Luke J. Sutherland, Hari Talreja

**Affiliations:** 1 Auckland City Hospital, Auckland, New Zealand; 2 Department of Renal Medicine, Middlemore Hospital, 100 Hospital Rd, Otahuhu, Auckland, 2025 New Zealand

**Keywords:** C3 glomerulonephritis, New Zealand, Chronic kidney disease

## Abstract

**Background:**

C3-glomerulonephritis can lead to progressive renal impairment from complement-mediated glomerular injury. Incidence and outcomes of C3-glomerulonephritis are not known in the New Zealand population.

**Methods:**

We reviewed all cases of C3-glomerulonephritis from the past 10 years at a tertiary referral centre in New Zealand. Descriptive information on baseline characteristics and clinical outcomes was collected.

**Results:**

Twenty-six patients were included (16 men; mean ± SD age 44 ± 25 years) with a median follow-up of 30 months. Disease incidence was 1.3 cases per million individuals, of which 42% were Pacific Islanders. Most patients presented with renal impairment, with a median (IQR) creatinine at diagnosis of 210 (146–300) μmol/L, and 11 (42%) patients presented with nephrotic syndrome. Seven (27%) patients progressed to end stage renal disease and 2 (8%) had died. End stage renal disease occurred in 20% of patients treated with immunosuppression and in 50% of those not treated. Complete remission was seen in 25% of patients treated with some form of immunosuppression and in 17% of those not treated.

**Conclusions:**

Our results are consistent with previous descriptions of C3-glomerulonephritis. There was a suggestion of better clinical outcomes in patients treated with immunosuppression. There was a higher disease incidence in Pacific Islanders, which may indicate an underlying susceptibility to complement dysfunction in this population.

## Background

C3-glomerulonephritis (C3GN) is a rare form of glomerulonephritis occurring in around 1–2 per million individuals [1–4]. The disease results from excessive activity of the C3-convertase in the alternative complement pathway, leading to downstream complement activation and glomerular injury from C3-deposition and the C5b-9 membrane attack complex (MAC) [5, 6]. Biopsies feature C3-dominant immunofluorescence of at least two orders of magnitude greater intensity than other immunofluorescence reactants [7]. C3GN can be distinguished from a related entity, dense deposit disease (DDD), based on the location of C3 deposits seen on electron microscopy. DDD features linear intramembranous deposits, whereas deposits in C3GN are mesangial, subendothelial or subepithelial [8].

The activity of the C3 convertase can be increased by several mechanisms. C3 nephritic factor (C3NeF) is an acquired autoantibody that stabilizes the C3 convertase. Functional factor H deficiency from an inherited mutation or acquired defect can result in loss of inhibition of the C3-convertase [1, 6]. Other complement proteins such as factors B and I can also be targeted by antibodies, and complement factor H-related protein gene mutations (CFHR1, CFHR2 and CFHR5) can also interfere with the binding of factor H to tissue-bound complement fragments [4, 8, 9].

C3GN patients have variable levels of proteinuria (which may be nephrotic-range) and microscopic or macroscopic hematuria [10, 11]. Reduced serum C3 levels are often seen. Patients have variable rates of renal function decline, and some may present with a rapidly progressive glomerulonephritis [9]. Most studies describe poor long term outcomes in patients with C3GN and frequent progression to end stage renal disease (ESRD) [11]. There are no previous descriptions of C3GN in New Zealand patients and treatment is based on international studies. New Zealand has an ethnically diverse population, and it is unknown whether Maori and Pacific Island populations have higher incidence of C3GN. To investigate this disease in our population, we reviewed all cases of C3GN over the past 10 years at our centre.

## Methods

This retrospective observational case series reviewed all patients with renal biopsy-confirmed C3GN over the past 10 years at Auckland City Hospital in Auckland, New Zealand. This is a tertiary referral centre providing renal histopathology services to around 1.9 million patients in the Auckland and Northland regions of New Zealand.

A database of renal histopathology reports was used to identify all cases with a final histological diagnosis of C3GN from January 1st, 2008 - December 31st, 2018. In addition, biopsies with a final diagnosis of membranoproliferative glomerulonephritis (prior to the widespread adoption of C3GN as a diagnosis) were reviewed with a renal histopathologist to re-evaluate whether they represented C3GN (C3-dominant immunofluorescence of at least two orders of magnitude greater intensity than other immunofluorescence reactants). All age groups were included. Patients with possible postinfectious GN based on electron microscopy findings were excluded if the clinical picture was supportive of this diagnosis. We excluded biopsies which featured C3 staining but had other histological or clinical features suggestive of an alternative diagnosis such as lupus nephritis or IgA nephropathy. All cases were reviewed with a renal histopathologist.

Baseline characteristics and clinical information were collected from electronic health records. Clinical outcomes included mortality, presence of nephrotic syndrome, incidence of ESRD (defined as requirement for maintenance dialysis or transplantation), renal survival (defined as absence of ESRD during follow-up), progressive disease (defined as doubling of serum creatinine), stable disease (defined as no doubling of creatinine but without complete remission) or complete remission. Complete remission was defined by an estimated glomerular filtration rate (eGFR) of > 60 ml/min/1.73 m^2^ (or a return to ±15% of baseline values if baseline eGFR was < 60 ml/min/1.73m^2^) and proteinuria < 0.5 g/24 h (or equivalent protein/creatinine ratio) [12, 13]. Demographic data included age, gender, ethnicity and comorbidities. Laboratory data included creatinine at diagnosis, C3/C4 levels, other complement testing (C3NeF testing by immunofixation, factor H deficiency, or other complement gene testing), proteinuria and hematuria. Nephrotic-range proteinuria was defined as a urine protein/creatinine ratio > 350 mg/mmol and microscopic hematuria was defined as > 5 red blood cells / high-power field. Treatment data included use of angiotensin converting enzyme (ACE) inhibitors, steroids and other immunosuppression. Histopathological findings included the pattern of disease on light microscopy, other features on light microscopy (crescents, scarring, interstitial infiltrate and interstitial fibrosis), immunofluorescence (IgG, IgM, IgA, C3, C1q) and electron microscopy (location of deposits).

Results were reviewed by a biostatistician. Continuous variables were expressed as mean +/− SD or median with IQR. Only a small number of patients were included therefore multivariate logistic regression was not performed. Study approval was obtained from the local ethics committees prior to study commencement.

## Results

Baseline characteristics are shown in Table 1. A total of 26 patients were identified (16 males; mean ± SD age 44 ± 25 years) with a median follow-up of 30 months. There were seven (27%) patients aged < 18 years. The most common ethnicity was Pacific Island (42%) followed by NZ European (31%) and NZ Maori (15%). The overall disease incidence was 1.3 cases per million individuals, 2.09 cases per million in NZ Maori and 5.6 cases per million in Pacific Island patients.

**Table 1:** Characteristics of patients at baseline and clinical presentation

Characteristic	All patients (***n*** = 26)
Age at diagnosis, years (SD)	44 (25)
Median follow-up, months (IQR)	30 (19–46)
Gender, no. male (%)	16 (62)
Ethnicity, no. (%)	
NZ European	8 (31)
Maori	4 (15)
Pacific Islander	11 (42)
Asian	3 (12)
Comorbidities, no. (%)	
Hypertension	14 (54)
Type 2 diabetes	4 (15)
Chronic kidney disease	4 (15)
Cr at diagnosis, umol/L (IQR)	210 (146–300)
Proteinuria, mg/mmol (IQR)	552 (293–983)
Nephrotic syndrome at diagnosis (%)	11 (42)
Dialysis at diagnosis, no. (%)	2 (8)
Normal renal function at diagnosis (%)	4 (15)
Hematuria, no. (%)	
Microscopic	25 (96)
Macroscopic	1 (4)
Complement levels, no. (%)	
Normal	8 (31)
Isolated low C3	12 (46)
Low C3 and C4	5 (19)
Not done	1 (4)
Complement screen, no. (%)	
Not done	11 (42)
C3NeF negative, Factor H normal	14 (54)
Factor H deficiency	1 (4)
Monoclonal gammopathy, no. (%)	
None	23 (88)
MGUS	1 (4)
Myeloma	2 (8)

*NZ New Zealand, *C3NeF* C3 nephritic factor, *MGUS* Monoclonal gammopathy of unknown significance

Most patients presented with renal impairment, with a median (IQR) creatinine at diagnosis of 210 (146–300) μmol/L, and nephrotic-range proteinuria (IQR) of 551.5 (293–983) mg/mmol (protein/creatinine ratio). Nephrotic syndrome was present in 11 (42%) patients. Twenty five (96%) patients had microscopic hematuria. Twelve (46%) patients had isolated low C3 and five (20%) had low C3 and C4. Fifteen (58%) patients had further complement screening but only one patient had an abnormality detected (Factor H deficiency detected on gene testing). Monoclonal gammopathy was seen in three (12%) patients (two with lambda light chain myeloma and one with MGUS).

The histopathological findings are summarized in Table 2. Eighteen (69%) of the biopsies had a membranoproliferative pattern on light microscopy, five (19%) had a diffuse proliferative pattern, two (8%) had a crescentic pattern and one (4%) a mesangial proliferative pattern. Apart from C3-dominant immunofluorescence, concurrent IgM staining was seen in 9 (35%) patients and IgG staining in 6 (23%) patients. IgA and C1q staining were less frequent.

**Table 2:** Histopathology characteristics on renal biopsy

Biopsy characteristic	All patients (***n*** = 26, (%)
Pattern on light microscopy	
Membranoproliferative	18 (69)
Diffuse proliferative	5 (19)
Crescentic	2 (8)
Mesangial proliferative	1 (4)
Immunofluorescent staining other than C3	
IgM	9 (35)
IgG	6 (23)
IgA	2 (8)
C1q	1 (4)
Kappa / lambda	0 (0)
Presence of EM deposits, no. (%)	
Subepithelial	17 (65)
Subendothelial	16 (62)
Mesangial	22 (85)
Intramembranous	9 (35)
Interstitial infiltrate, no. (%)	17 (65)
Intimal fibrosis, no. (%)	7 (27)
Scarring, median % (IQR)	7.5 (0–25)
Crescents, no. (%)	5 (19)

*EM Electron microscopy

Seventeen (65%) of the biopsies had some degree of interstitial infiltrate and median scarring (IQR) was only 7.5% (0–25). Deposits were seen in all locations on electron microscopy. Crescents were seen in five (19%) patients. No biopsies showed the typical dark, ribbon-like intramembranous deposits characteristics of DDD on electron microscopy.

Treatment and clinical outcomes are shown in Table 3. Two patients required dialysis at initial presentation, and overall seven (27%) had developed ESRD and two (8%) had died after a median follow-up of 30 months. Sixteen (62%) patients were treated with an ACE-inhibitor. Twelve patients were treated with prednisone and mycophenolate (MMF), three with prednisone and cyclophosphamide (of which one was also treated with plasma exchange), three with prednisone alone, and six patients received no immunosuppression. The two patients with myeloma were treated with a cyclophosphamide, bortezomib and dexamethasone (CyBorDex) regimen. One of these patients presented with nephrotic syndrome and was in remission from myeloma at 36 months follow-up, but had progressive renal impairment without a dialysis requirement. The second patient presented with macroscopic hematuria and subnephrotic proteinuria and had remission of both her myeloma and C3GN at 24 months follow-up. The patient with MGUS did not receive immunosuppression.

**Table 3:** Overall clinical outcomes

Treatment and outcome	All patients (***n*** = 26)
Occurrence of ESRD, no. (%)	7 (27)
Mortality, no. (%)	2 (8)
Renal survival, no. (%)	19 (73)
Stable disease, no. (%)	12 (46)
Progressive disease, no. (%)	5 (19)
Complete remission, no. (%)	6 (23)
Treatment and outcome, no. (%)	
ACE inhibitor	16 (62%)
Prednisone only	3 (12)
Prednisone and MMF	12 (46)
Other immunosuppression	5 (19)
Not treated	6 (23)

*ESRD End-stage renal disease, *ACE* Angiotensin converting enzyme

Clinical outcomes for each treatment subgroup are shown in Table 4. Of the 12 patients treated with prednisone and MMF, two had complete remission, seven had stable disease and three developed ESRD. Of the eight patients treated with other immunosuppression, three had complete remission, three had stable disease, one had progressive disease and one progressed to ESRD. Of the six patients not treated with immunosuppression one had complete remission, two had stable disease (of whom one died) and 3 developed ESRD (of whom one died). All patients with a diffuse proliferative or crescentic pattern on light microscopy received some form of immunosuppression.

**Table 4:** Clinical outcomes by treatment subgroup

Outcome	No treatment (***n*** = 6)	Prednisone and MMF (***n*** = 12)	Other immunosuppression (***n*** = 8)
Age at diagnosis, years (SD)	50 (22)	30 (20)	61 (18)
Gender, no. male (%)	3 (50)	8 (67)	5 (63)
Cr at diagnosis, umol/L (IQR)	225 (260)	165 (232)	215 (153)
Proteinuria, mg/mmol (IQR)	903 (895)	583 (687)	212 (584)
Dialysis at diagnosis, no. (%)	1 (4)	0 (0)	1 (4)
Patterns of disease on light microscopy (n)	MPGN (5), MsPGN (1)	MPGN (8), DPGN (4)	MPGN (5), DPGN (1), MsPGN (2)
Complete remission, no. (%)	1 (17)	2 (17)	3 (38)
Stable disease, no. (%)	2 (33)	7 (58)	3 (38)
Progressive disease, no.(%)	2 (17)	2 (17)	1 (13)
Occurrence of ESRD, no. (%)	3 (50)	3 (25)	1 (13)
Renal survival, no. (%)	3 (50)	9 (75)	7 (88)

*ESRD End-stage renal disease, *MMF* Mycophenolate mofetil, *MPGN* Membranoproliferative, *DPGN* Diffuse proliferative, *MsPGN* Mesangial proliferative

ESRD occurred in 20% of patients treated with some form of immunosuppression, and in 50% of those not treated. Complete remission was seen in 25% of patients treated with some form of immunosuppression and in 17% of those not treated. Renal survival occurred in 80% of those treated with some form of immunosuppression and in 50% of those not treated.

Of the 11 Pacific Island patients, nine (82%) were treated with some form of immunosuppression. Of those, one (9%) had complete remission, four (36%) had stable disease, and three (27%) presented with or developed ESRD. These results were similar to the overall group.

## Discussion

This study describes the characteristics of 26 patients with C3GN in the New Zealand population, with a median follow-up of 30 months. Characteristics of C3GN have not been previously described in this population. Cases were spread across all age groups and were more likely to be of Pacific Island ethnicity. Most patients presented with elevated creatinine and 42% of patients presented with nephrotic syndrome. An isolated low C3 was the most common finding on complement testing, although 20% had both low C3 and C4. The most common pattern seen on light microscopy was membranoproliferative. Crescents were seen on biopsy in 19% of patients. Three of the patients had myeloma or MGUS.

After a median observation time of 30 months, around a third of patients had progressed to ESRD, similar to previous descriptions [9]. ESRD occurred in 20% of patients treated with some form of immunosuppression, and in 50% of those not treated. Complete remission was seen in 25% of patients treated with some form of immunosuppression and in 17% of those not treated.

In a 2015 UK study of 80 patients with C3-glomerulopathy, 29% of patients progressed to ESRD after a median period of 28 months [11]. Other studies have found most patients eventually progress to ESRD within a decade of diagnosis [9, 14]. Studies by Medjeral-Thomas et al. and Sevais et al. found that immunosuppression failed to prevent C3GN from progressing to ESRD [9, 11].

In a 2015 Spanish retrospective observational study, 60 patients with C3 glomerulopathy were assessed for response to immunosuppression over a median period of 47 months [12]. Twenty patients did not receive immunosuppression, 22 received steroids and MMF, and 18 received other immunosuppression (steroids and cyclophosphamide or steroids alone). The incidence of ESRD was lower in treated versus untreated patients (3 out of 40 versus 7 out of 20). No patients in the steroids and MMF group had doubling of serum creatinine or developed ESRD. In patients who received steroids and MMF, renal survival was 100% at 5 years compared with 80% for those who received other immunosuppression and 72% in those not treated. The rates of remission were also higher in patients who received steroids and MMF.

There are no randomized trials to guide treatment decisions. Other treatments, such as plasma exchange and rituximab have only been discussed in case reports [15, 16]. Eculizumab, a monoclonal antibody widely used in complement-mediated HUS, has also been used in several case reports [15, 16]. Eculizumab acts on complement protein C5 to reduce the production of downstream complement factors which lead to glomerular injury in C3GN. Patients being considered for immunosuppression for treatment of C3GN in New Zealand usually receive prednisone and MMF. Prospective randomized trials would be useful to confirm the best treatment regimen.

Interestingly, we found only one underlying complement abnormality, although comprehensive complement testing was not performed in many cases. Previous reports state that up to 50% of cases of C3GN are caused by the presence of C3NeF [9]. This patient had factor H deficiency on gene testing and was treated with prednisone and MMF and had stable disease at follow-up. Initial screening available in New Zealand includes testing for C3NeF and Factor H deficiency. Testing for underlying complement pathway abnormalities may have particularly utility in those being considered for future renal transplantation [17]. Three of our cases had underlying monoclonal gammopathy, which has a well-established association with C3GN [18]. It is unknown whether Pacific Island patients have an underlying susceptibility to complement dysfunction which may account for the higher disease incidence in this population, or whether other novel mechanisms exist. Pacific Island patients have higher rates of diabetes, hypertension and obesity which result in higher rates of ESRD [19]. Aside from post-streptococcal glomerulonephritis, it is unknown whether Pacific Island patients are more susceptible to other forms of glomerulonephritis [20]. Pacific Island patients in our cohort did not appear to have significant differences in treatment or clinical outcomes.

The main limitation of this study is the small number of patients, however our data set represents all cases in our region of New Zealand over the past 10 years. Our histopathology laboratory serves around 1.9 million patients giving an estimated annual incidence of 1.3 per million, similar to that documented elsewhere [1]. We were not able to collect a sufficient number of patients to allow multivariate logistic regression to detect important predictors of renal outcome. As a result, any inferences of treatment efficacy are limited. Patients with more severe disease may have been more likely to receive immunosuppression. Although not performed in our centre, it has been suggested that C3GN patients with monoclonal gammopathy should undergo pronase immunofluorescence studies to rule out masked Ig deposits [21]. As noted, limited complement and genetic testing was available at our centre and this information could have implications on the treatment that patients are offered.

## Conclusion

This case series describes the clinical presentation of C3GN in New Zealand. Overall incidence appears to be similar to that reported in the international literature. We found a higher incidence in Pacific Island patients and that treatment with immunosuppression may reduce progression to ESRD in patients with C3GN.

## Data Availability

The datasets used and/or analysed during the current study are available from the corresponding author on reasonable request.

## Abbreviations

C3GN: C3 glomerulonephritis
DDD: Dense deposit disease
MAC: Membrane attack complex
C3NeF: C3 nephritic factor
ESRD: End stage renal disease
eGFR: Estimated glomerular filtration rate
MMF: Mycophenolate mofetil
NZ: New Zealand
